# Predictors of Poor Long-Term Outcomes in Patients with Newly Diagnosed Asymptomatic Cardiac Sarcoidosis: A Cardiovascular Magnetic Resonance Study

**DOI:** 10.3390/biomedicines13051093

**Published:** 2025-04-30

**Authors:** Nicoleta Nita, Dominik Felbel, Rima Melnic, Michael Paukovitsch, Wolfgang Rottbauer, Dominik Buckert, Johannes Mörike

**Affiliations:** Department of Internal Medicine II, University Medical Center, 89081 Ulm, Germany; dominik.felbel@uniklinik-ulm.de (D.F.); rima.melnic@uniklinik-ulm.de (R.M.); michael.paukovitsch@uniklinik-ulm.de (M.P.); wolfgang.rottbauer@uniklinik-ulm.de (W.R.); dominik.buckert@uniklinik-ulm.de (D.B.); johannes.moerike@uniklinik-ulm.de (J.M.)

**Keywords:** cardiac sarcoidosis, silent cardiac sarcoidosis, outcomes in cardiac sarcoidosis, cardiac magnetic resonance in cardiac sarcoidosis, right ventricular longitudinal strain in cardiac sarcoidosis, left ventricular late gadolinium enhancement in cardiac sarcoidosis

## Abstract

**Background:** The prevalence of patients with cardiac sarcoidosis (CS) diagnosed at a subclinical stage has increased; however, their long-term outcomes are not well known. **Objectives**: To investigate the incidence and predictors of adverse long-term outcomes in newly diagnosed patients with asymptomatic CS. **Methods**: Forty-three patients with newly diagnosed asymptomatic CS and comprehensive baseline evaluation with cardiovascular magnetic resonance (CMR) were studied. Asymptomatic CS was defined as CS in patients with biopsy-proven extracardiac sarcoidosis without cardiac symptoms but with abnormalities on CMR or positron emission tomography according to Heart Rhythm Society criteria. The primary endpoint was a composite of all-cause mortality, new ventricular arrhythmia or an atrioventricular block requiring cardiac device implantation, and hospitalization for heart failure. **Results**: Patients had a mean age of 56 ± 11 years and presented with normal left ventricular (LV) ejection fraction (58 ± 4%). A total of 44.2% of patients reached the composite endpoint during 5 years of follow-up. Patients with the primary endpoint were predominantly female (73.7%) and had a significantly higher prevalence of right ventricular (RV) involvement compared to patients without the primary endpoint (RV late gadolinium enhancement (LGE) in 26.3% vs. 4.2%, *p* = 0.037). In multivariate regression analysis, extensive LV LGE (HR 1.61, 95% CI 1.16–2.04, *p* = 0.004) and impaired RV global longitudinal strain (GLS) at baseline (HR 0.46, 95% CI 0.24–0.68, *p* = 0.015) were significantly predictive of the primary endpoint, whereas treatment with corticosteroids after CS diagnosis was significantly associated with improved outcomes (HR 7.69, 95% CI 1.11–11.11, *p* = 0.044). **Conclusions**: Newly diagnosed patients with asymptomatic CS have a significant incidence of adverse outcomes after 5 years of follow-up. The extent of LV LGE and impaired RV GLS at baseline predict poor long-term outcomes in asymptomatic CS.

## 1. Introduction

Cardiac sarcoidosis (CS) is an autoinflammatory cardiomyopathy characterized by granulomatous infiltration of the myocardium [1]. Patients with CS may be clinically silent or experience sudden cardiac death, with most patients presenting with ventricular arrhythmias, high-grade atrioventricular block, and heart failure due to biventricular dysfunction [2]. Accurate diagnosis of CS is challenging as a result of the lack of randomized trials, and current diagnostic and therapeutic strategies are based on consensus recommendations and are not universally adopted [3]. Cardiovascular magnetic resonance (CMR) is a key imaging modality in CS, and the presence of myocardial late gadolinium enhancement (LGE) constitutes a major diagnostic criterion for CS [2], as CMR studies proved excellent diagnostic performance and high negative predictive value [4]. However, CMR interpretation may be subject to interreader interpretation in inconclusive cases, and positron emission tomography (PET) performed in conjunction with CMR allows for diagnostic confirmation in patients with CS [3,5]. As a consequence, clinically manifested CS represents merely the tip of an iceberg, as multimodality imaging techniques have revealed that cardiac involvement is detected four to five times more frequently than what is clinically apparent [6,7]. However, previous research has focused on patients with clinically manifest CS, and long-term outcomes in patients with CS diagnosed at an asymptomatic stage have been poorly investigated.

The purpose of this study was to investigate long-term outcomes in patients with newly diagnosed asymptomatic CS and to determine predictors of poor outcomes, which could optimize risk stratification and management at an earlier stage of the disease.

## 2. Materials and Methods

### 2.1. Study Population

A total of 43 consecutive patients with cardiac sarcoidosis were identified from a larger cohort of patients with extracardiac sarcoidosis who were referred to the Heart and Lung Department of the University Hospital of Ulm from 2000 to 2021. All patients with CS (diagnosed according to the Heart Rhythm Society criteria [8]) without cardiac symptoms, with available CMR examination at the time of diagnosis, and with extensive follow-up were included in the current analysis. Asymptomatic CS was defined as CS in patients with biopsy-proven extracardiac sarcoidosis with no cardiac symptoms but abnormalities on screening electrocardiogram (ECG) or echocardiography and typical findings for CS on imaging using cardiovascular magnetic resonance (CMR) or positron emission tomography (PET). Typical CMR abnormalities indicative of CS included the presence of left ventricular (LV) late gadolinium enhancement (LGE) with a non-ischemic pattern and common locations for CS such as basal subepicardial septal, multifocal septal with or without LGE involvement of the right ventricular (RV) septum, or RV free wall according to current recommendations of the American Heart Association [9]. All patients included in this analysis had a significant burden of LGE located septal subepicardial or septal intramural multifocal. RV involvement was defined as LGE within the RV free wall.

Patients with cardiac symptoms at the time of diagnosis, such as heart failure symptoms, symptomatic arrhythmia, or symptomatic high-grade block, were excluded from the study.

Demographic and clinical data, medical history, and follow-up data, including death and arrhythmic events, were retrospectively collected from the electronic medical records of the University Hospital of Ulm. The median follow-up time was 5.3 years from the CMR examination. The endpoint of interest was the composite of all-cause death, sustained ventricular tachycardia requiring defibrillator implantation, advanced atrioventricular block requiring pacemaker implantation, and hospitalization for heart failure.

Arrhythmic events were documented using 12-lead ECG and Holter monitors.

This study was approved by the ethical board of the University of Ulm (approval number 238/16) and complied with the Declaration of Helsinki.

### 2.2. CMR Protocol

Patients underwent CMR examination using a protocol designed for inflammatory cardiac disease on a clinical 1.5 Tesla scanner (Achieva, Philips Healthcare, Best, The Netherlands). Multi-slice b-SSFP Cartesian sequences and retrospective ECG gating were performed to cover the entire cardiac cycle with 32 phases and following acquisition parameters: temporal resolution of 30 ms, repetition time 2.42 ms, echo time 1.2 ms, flip angle 60°, field of view 380 × 380 mm^2^, in plane resolution 14 × 1.4 mm^2^, slice thickness of 8 mm. Ventricular LGE acquisitions were performed in all subjects using an extracellular contrast agent (Dotarem^®^, Guerbet, Villepinte, France) and segmented inversion-recovery gradient sequences in the long and short axis according to the recommendations of the society of Cardiac magnetic resonance [10]. The study protocol for 21 recent patients included a modified look-locker sequence in a 5(3)3 scheme for T1 mapping before and after the application of the contrast agent. The T2 maps were obtained using a gradient spin-echo sequence.

All CMR measurements, including volumetry, ventricular strain (LV and RV free wall GLS), parametric mapping, and LGE assessment, were performed using the software CVI42, v6.0 (Circle Cardiovascular Imaging Inc., Calgary, AB, Canada).

### 2.3. Reproducibility Analysis

The interobserver reproducibility of strain measurements was tested in 25 randomly selected individuals and was found to be good (Kappa coefficient 0.72).

### 2.4. Statistics

Statistical analyses were performed using IBM SPSS Statistics, version 29.0 (IBM Corporation, Armonk, NY, USA). Continuous variables with normal distribution were expressed as mean ± standard deviation (SD) or as median with interquartile range (IQR) in the case of non-normal distribution. Categorical variables were presented as absolute numbers and percentages. Comparisons between subgroups were performed using a *t*-test or Mann–Whitney U test for continuous variables or using the Chi square test for categorical variables. The area under the curve (AUC) was determined through receiver-operating characteristic (ROC) analysis to determine whether imaging parameters would predict the combined endpoint. Optimal cut-off values for relevant parameters with an AUC above 0.7 were generated from the ROC analysis using the Youden threshold. The cumulative incidence of adverse events was estimated using Kaplan–Meier analysis, and Log-rank tests were performed to assess differences between subgroups. Multivariable Cox regression analysis was performed to assess the influence of relevant parameters on AF. The algorithm was applied to all potentially relevant variables, including parameters from univariate logistic regression analysis with *p* <  0.10, and each model included a maximum of three potential predictors, given the reduced number of events [11]. Collinearity between parameters was analyzed using variance inflation factors. A two-tailed *p* < 0.05 was considered statistically significant.

## 3. Results

### 3.1. Patient Characteristics

The study included 43 patients with asymptomatic CS. Seven patients (16.3%) had definite CS based on histological confirmation by endomyocardial biopsy, whereas 83.7% of patients had probable CS based on positive extracardiac biopsy and typical imaging abnormalities on PET and CMR. Endomyocardial biopsy in the seven patients mentioned was performed at the clinician’s discretion, mostly due to a lower extent of late gadolinium enhancement (% of LV mass) despite a typical CS location of LGE (septal, subepicardial, or intramural multifocal). One patient had isolated CS without extracardiac involvement. Diagnostic work-up with CMR was initiated in this patient because of asymptomatic intermittent atrioventricular block on 12-lead ECG and reduced ventricular strain on echocardiography during a routine check-up. After the documentation of typical CS abnormalities on CMR, CS was confirmed in this patient through endomyocardial biopsy. Table 1 summarizes the baseline demographic, clinical, and laboratory data. The mean age was 56 ± 11 years, and 58.1% were women. Patients who reached the endpoint were older (59 ± 12 vs. 54 ± 9, *p* = 0.085), predominantly female (73.7% vs. 45.8, *p* = 0.066), and had a significantly lower glomerular filtration rate (GFR) compared to patients who did not reach the endpoint. Levels of cardiac markers, including NT-proBNP and Troponin T, and the prevalence of comorbidities were similar between patients who reached the endpoint and those who did not.

### 3.2. Results of Multimodality Imaging

**Figure 1:**
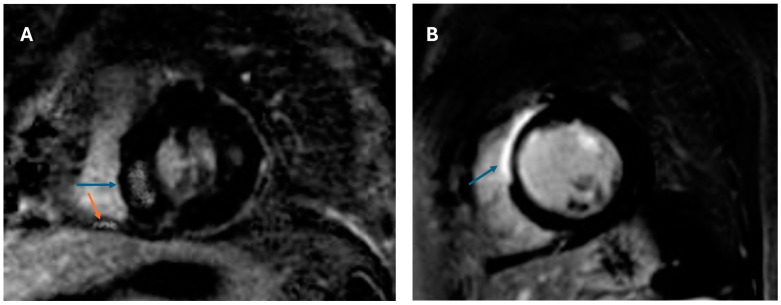
CMR-LGE findings in asymptomatic patients with cardiac sarcoidosis and histological confirmation: (**A**) Patient with hypertrophic phenotype and patchy distribution of LGE in the LV (blue arrow) and inferior wall of the RV (orange arrow). (**B**) Asymptomatic patient with extensive LV LGE (14% of LV mass). CMR = cardiovascular magnetic resonance; LGE = late gadolinium enhancement; LV = left ventricle; RV = right ventricle.

Table 2 shows the results of cardiac imaging with CMR and PET-CT at baseline. All patients had LV LGE consistent with CS, and 77.8% had ventricular ^18^F-FDG uptake, with similar prevalences between patients who reached the endpoint and those who did not. The prevalence of RV LGE at baseline was significantly higher in patients who reached the cumulative endpoint during follow-up (26.3% vs. 4.2%, *p* = 0.037), as shown in Figure 1. In the entire cohort, patients had normal biventricular function (LVEF 58 ± 4%, RVEF 57 ± 4%), with no significant differences between patients who reached the study endpoint and those who did not. The extent of LV LGE was significantly higher in patients who reached the endpoint compared to those who did not (14 ± 3% vs. 11 ± 3%, *p* < 0.001). In CMR feature tracking analysis, RVFW GLS was significantly lower in patients who reached the endpoint compared to those who did not (−19.6 ± 2.2% vs. −21.7 ± 1.7, *p* < 0.001), whereas LV GLS did not differ significantly between subgroups (−12.6 ± 1.2% vs. −13.2 ± 1.5, *p* = 0.142). Parametric mapping analysis showed significantly lower global native T1 and global ECV at baseline in patients with endpoint, whereas global T2 mapping was similar between patients who reached the endpoint and those who did not.

### 3.3. Clinical Outcomes

Twenty-five patients (58.1%) in the entire cohort received immunosuppressive therapy with corticosteroids after CS diagnosis, and the proportion of patients treated with corticosteroids after CS diagnosis was significantly lower in patients who reached the study endpoint (36.8% vs. 75%, *p* = 0.012). There were no significant differences in baseline characteristics, including imaging variables, between patients treated with corticosteroids and those not treated with corticosteroids, except for hypertension, which was more prevalent in patients treated with corticosteroids, as shown in Table S1. Over a follow-up period of 5 years, 19 patients (44.2%) reached the composite endpoint. Two patients died (median event time 48 months), fourteen patients (32.6%) developed high-grade atrioventricular block at a median event time of 37 months, and sustained ventricular tachycardia was documented in eight patients (18.6%, median event time of 35 months). All patients who reached the composite endpoint received an intracardiac device, either a pacemaker or a defibrillator, after a median event time of 38 months. Hospitalization for heart failure was documented in five patients at a median event time of 35 months, all of whom had previously experienced sustained VT.

### 3.4. Predictors of Adverse Events

**Figure 2:**
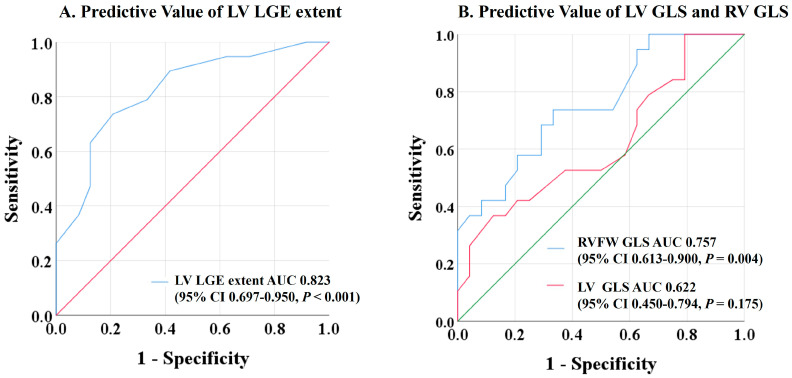
Receiver-operating characteristic curves to detect patients with major adverse cardiovascular events. LV LGE extent and RVFW GLS showed the highest areas under the curve among the CMR markers of ventricular function at baseline. LV GLS was not predictive of outcomes. CMR = cardiovascular magnetic resonance; LV GLS = left ventricular global longitudinal strain; LV LGE = left ventricular late gadolinium extent; RVFW GLS = right ventricular free wall global longitudinal strain.

In the ROC analysis, the extent of LV LGE and RVFW GLS showed the best predictive value for adverse events at follow-up, whereas LV GLS was not associated with adverse outcomes (Figure 2). Optimized cut-off values of 12.5% for LV LGE extent and −21.3% for RVFW GLS were identified as predictive of adverse events.

Table 3 and Table 4 summarize the results of univariate and multivariate Cox regression analysis, including cardiac imaging and clinical variables.

In multivariable Cox regression analysis, none of the clinical, laboratory, and demographic variables, including age and gender, reached statistical significance for the incidence of adverse events during follow-up. Although statistically significant in univariate analysis, GFR did not reach significance in multivariable regression analysis. High LV LGE extent (HR 1.61, 95% CI 1.16–2.04, *p* = 0.004) and reduced RVFW GLS (HR 0.46, 95% CI 0.24–0.68, *p* = 0.044) predicted poor outcomes in patients with asymptomatic CS, whereas corticosteroid therapy after CS diagnosis was independently associated with better outcomes (HR 7.69, 95% CI 1.11–11.11, *p* = 0.044).

**Figure 3:**
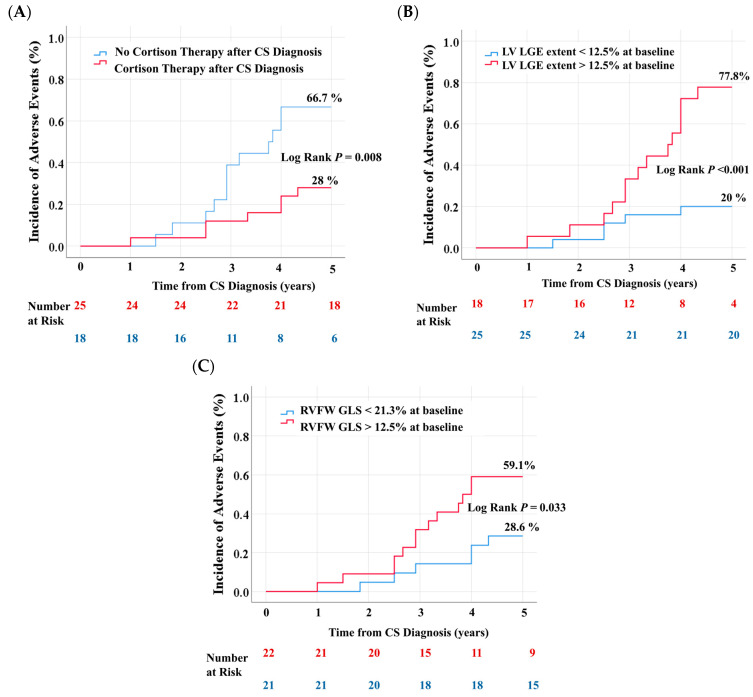
Cumulative incidence of adverse events in patients with asymptomatic CS. Kaplan–Meier Curves stratified using cortison therapy after CS diagnosis (**A**), extent of LV LGE (**B**), and RVFW-GLS (**C**). LV LGE = left ventricular late gadolinium enhancement; RVFW GLS = right ventricular free wall global longitudinal strain.

Figure 3 shows the cumulative incidence of adverse outcomes over a follow-up period of 5 years, after subsequent stratification of the cohort by the presence of corticosteroid therapy after CS diagnosis, the presence of extensive LV LGE, and the presence of reduced RVFW GLS. Patients treated with corticosteroids after CS diagnosis had a significantly lower incidence of adverse outcomes during follow-up compared with patients not treated with corticosteroids (28% vs. 66.7%, Log Rank *p* = 0.008, Figure 3A). The incidence of adverse outcomes was significantly higher in patients with extensive LV LGE (77.8% vs. 20%, Log Rank *p* < 0.001, Figure 3B) or reduced RVFW GLS (59.1% vs. 28.6%, Log Rank *p* = 0.033, Figure 3C) at baseline.

## 4. Discussion

The present study showed that patients diagnosed with asymptomatic CS have a significant cumulative risk of 42.5% for future adverse events at five years from diagnosis.

It is unknown whether patients with clinically silent CS have a better prognosis than patients with clinically manifest CS, as previous research has mainly studied patients diagnosed with symptomatic CS [12,13]. However, autopsy studies have documented a significant risk of sudden cardiac death in patients with CS but no previous cardiac symptoms [14]. We report a 5-year overall survival rate of 95.3%, which is slightly higher than the 90% 5-year survival rate in patients with clinically manifest CS reported in larger contemporary studies [15,16]. Patients with newly diagnosed asymptomatic CS in our study had a cumulative incidence of sustained ventricular tachycardia at 5 years of 18.6%, which is comparable to the incidence in most cohorts of clinically manifest CS, including the multicenter Illuminate CS-Registry of 512 patients [16]. Evidence of both late gadolinium enhancement on CMR or abnormal uptake on FDG-PET has been associated with an increased risk of VT and cardiovascular death, independent of symptoms at the time of diagnosis [17]. Sustained VT in patients with overt CS predisposes them to poor outcomes, as shown by Kusano et al. [18]. Our results confirm these findings, as 62.5% of the study patients who developed sustained ventricular tachycardia requiring defibrillator implantation were subsequently hospitalized for heart failure. Therefore, the significant incidence of VT in this cohort with silent CS underlines the importance of careful screening for ventricular arrhythmias even in asymptomatic patients with CS.

### 4.1. Predictors of Adverse Outcomes

The extent of LV LGE and the presence of impaired RVFW GLS independently predicted adverse events over a long-term period of 5 years in this study cohort of asymptomatic patients with CS and preserved left and right ejection fractions. The predictive value of extensive LV LGE on CMR has been demonstrated in previous studies in patients with clinically manifest CS and reduced LVEF [19]; however, its prognostic significance in patients with asymptomatic CS and normal LVEF is unclear. We report a significant extent of myocardial LGE of 12 ± 4 % of LV mass in a cohort with normal LVEF, which, although lower than in patients with clinically manifest CS in previous studies [20], was superior to all other CMR variables in predicting the incidence of adverse events. The predictive value of RV function had also been suggested in previous works [21,22,23], revealing that the RV dysfunction and inflammatory involvement of the RV wall, shown by CMR or nuclear imaging, predicted life-threatening arrhythmias in CS patients. In addition, Albakaa et al. demonstrated that reduced RVFW GLS predicted poor outcomes in a contemporary cohort of 51 patients with clinically manifest CS and reduced RVEF [24]. Our findings support these data but also extend the knowledge by identifying impaired RV longitudinal strain caused by CMR as an important predictor of poorer outcomes in asymptomatic patients with newly diagnosed CS. Current guidelines are inconsistent regarding the management of silent CS, as the impact of treatment in asymptomatic CS is not completely understood [9]. Our observation of a lower incidence of adverse events in patients with asymptomatic CS treated with corticosteroids after CS diagnosis suggests that the timely initiation of corticosteroid therapy at an earlier stage of the disease may prevent extensive ventricular scarring and dysfunction. Whether corticosteroids improve the prognosis in asymptomatic patients with CS cannot be concluded from the data available today, and larger studies are needed to confirm our findings.

### 4.2. Strengths and Limitations

This study had several limitations. First, it was a retrospective, observational study with a small population, conducted in a tertiary referral center. However, compared with previous research, our study is the only one to analyze long-term outcomes using time-to-event data in asymptomatic patients with newly diagnosed CS and normal biventricular ejection. Parametric mapping variables, although prognostically relevant in various cardiomyopathies, were not included in the multivariable regression analysis, given that parametric analysis was available in a reduced number of patients.

## 5. Conclusions

Patients with newly diagnosed asymptomatic CS present a 44.2% risk of adverse events during the first 5 years of follow-up. Despite being asymptomatic and with normal biventricular ejection fractions, CMR assessment with LGE, parametric mapping, and global longitudinal strain identified significant left and right ventricular involvement. The extent of LV LGE and the presence of impaired RV LGE at baseline were identified as significant predictors of poor outcome and may help clinicians to guide management and risk stratification in such patients. The potential of corticosteroids to improve long-term outcomes in asymptomatic CS should be investigated in larger studies.

## Figures and Tables

**Table 1:** Baseline characteristics.

	No Event (n = 24)	Event (n = 19)	*p* Value
Age	54 ± 9	59 ± 12	0.085
Female	11 (45.8)	14 (73.7)	0.066
Body Mass Index	1.9 ± 0.2	1.9 ± 0.3	0.765
Comorbidities			
Hypertension	7 (29.2)	5 (26.3)	0.836
Diabetes Mellitus	1 (4.2)	2 (10.5)	0.416
Coronary artery disease	2 (8.3)	0 (0)	0.198
Sleep apnea	1 (4.2)	3 (15.8)	0.193
Definite diagnosis	4 (16.7)	3 (15.8)	0.938
Probable diagnosis	20 (83.3)	16 (84.2)	0.938
Extracardiac sarcoidosis	23 (95.8)	19 (100)	0.368
Extracardiac organ involvement			
Lung	22 (91.7)	18 (94.7)	0.695
Lymph Node	10 (41.7)	7 (36.8)	0.748
Skin	3 (12.5)	3 (15.8)	0.757
Laboratory values			
Troponin T (pg/mL)	8 ± 3	10 ± 3	0.182
NT-proBNP (ng/L)	291 ± 156	355 ± 129	0.160
GFR (mL/min)	81 ± 14	69 ± 121	0.030

Values are n (%) or mean ± SD. NT-proBNP = N-terminal pro B-type natriuretic peptide. GFR = glomerular filtration rate.

**Table 2:** Results of multimodality imaging.

	No Event (n = 24)	Event (n = 19)	*p* Value
CMR			
LVEDVI, mL/m^2^	90 ± 13	89 ± 13	0.876
LVEF %	58 ± 4	57 ± 2	0.424
LV Mass g/m^2^	62 ± 15	62 ± 14	0.923
LGE extent, % of LV mass	11 ± 3	14 ± 3	<0.001
RVEDVI, mL/m^2^	86 ± 12	85 ± 14	0.857
RVEF %	58 ± 3	56 ± 4	0.230
RV LGE	1 (4.2)	5 (26.3)	0.037
RVFW-GLS (%)	−21.7 ± 1.7	−19.6 ± 2.2	0.001
LV-GLS (%)	−13.2 ± 1.5	−12.6 ± 1.2	0.142
Global Native T1, ms ^a^	1078 ± 36	1166 ± 85	0.021
Global ECV	27.4 ± 2.5	31 ± 3.4	0.019
Global T2, ms	55 ± 8	57 ± 5	0.390
PET ^b^			
Ventricular ^18^F-FDG uptake	6/7 (85.7)	8/11 (72.7)	0.518

Values are n (%) or mean ± SD. CMR = cardiac magnetic resonance; ECV = extracellular volume; ^18^F-FDG = ^18^F-Fluorodeoxyglucose; GLS = global longitudinal strain; LGE = late gadolinium enhancement; LVEDVI = left ventricular end diastolic volume index; LVEF = left ventricular ejection fraction; PET = positron emission tomography; RVEDVI = right ventricular end diastolic volume index; RVEF = right ventricular ejection fraction; RVFW = right ventricular free wall; ^a^ parametric mapping was available for 21 patients. ^b^ PET was available for 18 patients.

**Table 3:** Results of univariate Cox regression analyses on demographics, laboratory, and imaging variables for the prediction of adverse events in patients with asymptomatic CS.

	HR (95% CI)	*p* Value
Demographics/Comorbidities		
Age	1.05 (0.99–1.12)	0.091
Gender	3.30 (0.90–12.13)	0.071
Hypertension	1.15 (0.30–4.43)	0.836
Lack of cortison therapy after CS diagnosis	5.14 (1.38–19.10)	0.014
Diagnosis through myocardial biopsy	0.938 (0.183–4.80)	0.938
Laboratory Variables		
NT-proBNP (ng/mL)	1.003(0.999–1.007)	0.159
Troponin (pg/mL)	1.61 (0.93–1.44)	0.182
GFR (mL/min)	0.96 (0.92–0.99)	0.042
Imaging Variables		
LVEDVI, mL/m^2^	0.99 (0.95–1.05)	0.873
RVEDVI, mL/m^2^	0.99 (0.95–1.04)	0.853
RVFW-GLS (%)	0.57 (0.38–0.84)	0.005
LV-GLS (%)	0.69 (0.43–1.13)	0.147
Ventricular ^18^F-FDG uptake	2.25 (0.18–27.36)	0.525
Global Native T1 Mapping, ms	1.02 (0.99–1.04)	0.058
Global ECV (%)	1.49 (1.05–2.20)	0.042
Global T2, ms	1.08 (0.91–1.28)	0.369
LVEF (%)	0.93 (0.78–1.10)	0.418
LV LGE extent (%)	1.54 (1.18–2.02)	0.002
LV Mass, g/m^2^	0.99 (0.96–1.04)	0.921
RVEF (%)	0.90 (0.76–1.07)	0.229
RV LGE	8.21 (0.68–77.77)	0.066

CS = cardiac sarcoidosis; ECV = extracellular volume; 18F-FDG = 18F-Fluorodeoxyglucose; GFR = glomerular filtration rate; GLS = global longitudinal strain; LGE = late gadolinium enhancement; LVEDVI = left ventricular end diastolic volume index; LVEF = left ventricular ejection fraction; RVEDVI = right ventricular end diastolic volume index; RVEF = right ventricular ejection fraction; LV LGE = left ventricular late gadolinium enhancement; NT-proBNP = N-terminal pro B-type natriuretic peptide; RVFW GLS = right ventricular free wall global longitudinal strain.

**Table 4:** Multivariable predictors of adverse events in patients with asymptomatic cardiac sarcoidosis.

	Model 1 (n = 43, e = 19)		Model 2 (n = 43, e = 19)		Model 3 (n = 43, e = 19)		Model 4 (n = 43, e = 19)	
	HR (95% CI)	*p* Value	HR (95% CI)	*p* Value	HR (95% CI)	*p* Value	HR (95% CI)	*p* Value
Age	1.08 (0.99–1.18)	0.077	-	N/A	1.07 (0.99–17)	0.083	-	N/A
Cortison	-	N/A	-	N/A	4.76 (1.04–13.30)	0.050	7.69 (1.11–11.11)	0.044
GFR	-	N/A	0.95 (0.89–1.01)	0.106	-	N/A	-	N/A
RVFW GLS (%)	0.46 (0.24–0.68)	0.015	0.56 (0.32–0.89)	0.044	-	N/A	0.39 (0.19–0.83)	0.014
LV LGE (%)	1.61 (1.16–2.04)	0.004	1.65 (1.17–2.13)	0.004	1.55 (1.13–2.12)	0.007	1.58 (1.14–2.08)	0.006

Multivariable models based on cause-specific Cox regression analyses. e = number of events; GFR = glomerular filtration rate; LV LGE = left ventricular late gadolinium enhancement; n = number of patients; N/A not applicable; RVFW GLS = right ventricular free wall global longitudinal strain.

## Data Availability

The data underlying this article will be shared upon reasonable request to the corresponding author. The data are not publicly available due to data privacy laws.

## Supplementary Materials

The following supporting information can be downloaded at: https://www.mdpi.com/article/10.3390/biomedicines13051093/s1, Table S1. Baseline Characteristics stratified by Cortison therapy after CS diagnosis.

## Abbreviations

The following abbreviations are used in this manuscript:

AUC	Area under the curve
CMR	Cardiovascular magnetic resonance
CS	Cardiac sarcoidosis
GLS	Global longitudinal strain
LV	Left ventricle
LVEF	Left ventricular ejection fraction
LGE	Late gadolinium enhancement
PET	Positron emission tomography
ROC	Receiver-operating characteristic
RV	Right ventricle
RVFW	Right ventricular free wall
VT	Ventricular tachycardia
