# Supplementary material for: Predictors of Poor Long-Term Outcomes in Patients with Newly Diagnosed Asymptomatic Cardiac Sarcoidosis: A Cardiovascular Magnetic Resonance Study
Source: Biomedicines. 2025 Apr 30;13(5):1093. doi: 10.3390/biomedicines13051093 (PMC12109307; doi:10.3390/biomedicines13051093)
Supplement: Supplementary file 1 [file biomedicines-13-01093-s001.zip › biomedicines-3573854-supplementary.pdf]

Article

Predictors of Poor Long-Term Outcomes in Patients with Newly Diagnosed Asymptomatic Cardiac Sarcoidosis: A Cardiovascular Magnetic Resonance Study

Nicoleta Nita *, Dominik Felbel, Rima Melnic, Michael Paukovitsch, Wolfgang Rottbauer, Dominik Buckert and Johannes Mörike

Department of Internal Medicine II, University Medical Center, 89081 Ulm, Germany; dominik.felbel@uniklinik-ulm.de (D.F.); rima.melnic@uniklinik-ulm.de (R.M.); michael.paukovitsch@uniklinik-ulm.de (M.P.); wolfgang.rottbauer@uniklinik-ulm.de (W.R.); dominik.buckert@uniklinik-ulm.de (D.B.); johannes.moerike@uniklinik-ulm.de (J.M.)

* Correspondence: nicoleta.nita@uniklinik-ulm.de; Tel.: +4973145120

Supplementary Table S1. Baseline Characteristics stratified by Cortison therapy after CS diagnosis

	No Cortison Therapy (n=18)	Cortison Therapy (n=25)	*P* Value
Age	56±11	56±10	0.831
Female	12(45.8)	13 (52)	0.336
Comorbidities			
Hypertension	2(11.1)	10(40)	0.037
Diabetes Mellitus	2(11.1)	1(4)	0.367
Coronary artery disease	0(0)	2(8)	0.219
Laboratory values			
NT-pro BNP (ng/l)	349±117	299±163	0.278
GFR (ml/min)	70±19	79±17	0.096
**Imaging variables**			
LVEF %	59±3	57±4	0.084
LGE extent, % of LV mass	13±3	12±4	0.122
RVEF %	57±3	56±4	0.725
RV LGE	2(11.1)	4(16)	0.648
RVFW-GLS (%)	-20.5±2	-21±2.3	0.494
LV-GLS (%)	-12.9±1.3	-13±1.5	0.729
Global Native T1, ms	1155±98	1116±70	0.342
Global ECV	30.7±4.1	29.3±3.3	0.426
Global T2, ms	58±4	55±7	0.287
Ventricular ^{18}F-FDG uptake	5/8 (62.5)	9/10 (90)	0.163

Values are n (%) or mean± SD. CS = cardiac sarcoidosis; ECV = extracelular vlolume; 18F-FDG = 18F-Fluorodeoxyglucose; GLS = global longitudinal strain; GFR = glomerular filtration rate; LGE = late gadolinium enhancement; LVEF = left ventricular ejection fraction; NT-proBNP = N-terminal pro B-type

natriuretic peptide; PET = positron emission tomography; RVEF = right ventricular ejection fraction; RVFW= right ventricular free wall;
